# Proteomics reveals specific biological changes induced by the normothermic machine perfusion of donor kidneys with a significant up-regulation of Latexin

**DOI:** 10.1038/s41598-023-33194-z

**Published:** 2023-04-11

**Authors:** Gianluigi Zaza, Flavia Neri, Maurizio Bruschi, Simona Granata, Andrea Petretto, Martina Bartolucci, Caterina di Bella, Giovanni Candiano, Giovanni Stallone, Loreto Gesualdo, Lucrezia Furian

**Affiliations:** 1 Nephrology, Dialysis and Transplantation Unit, Department of Medical and Surgical Sciences, University-Hospital of Foggia, Via L. Pinto 1, 71122 Foggia, Italy; 2 Kidney and Pancreas Transplantation Unit, University of Padua, Padua, Italy; 3 Laboratory of Molecular Nephrology, IRCCS Istituto Giannina Gaslini, Genoa, Italy; 4 Department of Experimental Medicine (DIMES), University of Genoa, Genoa, Italy; 5 Core Facilities - Proteomica E Metabolomica Clinica, IRCCS Istituto Giannina Gaslini, Genova, Italy; 6 Nephrology, Dialysis and Transplantation Unit, Department of Precision and Regenerative Medicine and Ionian Area (DiMePRe-J), University of Bari “Aldo Moro”, Bari, Italy

**Keywords:** Nephrology, Kidney

## Abstract

Renal normothermic machine perfusion (NMP) is an organ preservation method based on the circulation of a warm (35–37 °C) perfusion solution through the renal vasculature to deliver oxygen and nutrients. However, its biological effects on marginal kidneys are unclear. We therefore used mass spectrometry to determine the proteomic profile of kidney tissue and urine from eight organs reconditioned for 120 min using a Kidney Assist device. Biopsies were taken during the pre-implantation histological evaluation (T-1), at the start of back table preparation (T0), and after 60 and 120 min of perfusion (T60, T120). Urine samples were collected at T0 (urine produced in the first 15 min after the beginning of normothermic reperfusion), T30, T60 and T120. Multiple algorithms, support vector machine learning and partial least squares discriminant analysis were used to select the most discriminative proteins during NMP. Statistical analysis revealed the upregulation of 169 proteins and the downregulation of 196 during NMP. Machine learning algorithms identified the top 50 most discriminative proteins, five of which were concomitantly upregulated (LXN, ETFB, NUDT3, CYCS and UQCRC1) and six downregulated (CFHR3, C1S, CFI, KNG1, SERPINC1 and F9) in the kidney and urine after NMP. Latexin (LXN), an endogenous carboxypeptidase inhibitor, resulted the most-upregulated protein at T120, and this result was confirmed by ELISA. In addition, functional analysis revealed that the most strongly upregulated proteins were involved in the oxidative phosphorylation system and ATP synthesis, whereas the downregulated proteins represented the complement system and coagulation cascade. Our proteomic analysis demonstrated that even brief periods of NMP induce remarkable metabolic and biochemical changes in marginal organs, which supports the use of this promising technique in the clinic.

## Introduction

Kidney transplantation is the best treatment for end-stage renal disease, but the availability of healthy donor organs is limited^1^. Many patients on the waiting list must therefore accept kidneys from extended criteria donors (ECDs)^2^ or donation after circulatory death (DCD)^3^. These kidneys, from older, comorbid and ischemic donors, are more prone to severe functional alterations and take longer to regain function (delayed graft function) than kidneys from younger, standard criteria donors (SCDs) due to the effects of ischemia/reperfusion (I/R) injury^4^. This complication enhances the risk of early immunological alterations, including acute rejections^5^.

Ischemic effects can be prevented by hypothermic machine perfusion, which is standard clinical practice for the safe transport of kidneys from the donor to the recipient center. This has a better outcome than static cold storage^6^, although the condition of the kidney gradually deteriorates in an anaerobic environment^7^. To avoid such deterioration, normothermic machine perfusion (NMP) involves the circulation of a warm (35–37 °C) perfusion solution through the renal vasculature to deliver oxygen and nutrients, thus restoring cellular metabolism and replenishing the pool of adenosine triphosphate (ATP)^8^. Under optimal conditions, this can prevent organ deterioration and promote recovery. By restoring kidney function, NMP also provides an opportunity to assess organ quality before transplantation and facilitates the delivery of pre-transplant therapies^8^. However, the overall biological impact of this technology on the kidney is only partially understood.

One way to address the knowledge gap is the application of comparative proteomics. For example, mass spectrometry was used in one recent study to show that NMP with urine recirculation preserves and revitalizes kidney metabolism, primarily by reducing the abundance of damage-associated molecular patterns (DAMPs), improving mitochondrial glucose metabolism, and minimizing biochemical alterations induced by I/R injury^9^. Furthermore, nanoparticle tracking analysis demonstrated that discarded ECD kidney grafts release distinct extracellular vesicles (EVs) during NMP that may be suitable for the assessment of kidney graft quality^10^.

We therefore applied an innovative comparative mass spectrometry approach to measure acute changes in the protein content in kidney tissue and urine obtained from discarded kidneys (after histological evaluation) undergoing NMP. The analysis may indicate NMP-induced changes in kidney functions and determine the molecular effects of this procedure.

The proteins that are modulated by NMP may be developed in the future as early urinary biomarkers and/or new therapeutic targets.

## Results

### Description of the perfusion process

Eight kidneys deemed unsuitable for transplantation underwent NMP treatment between April 2020 and April 2021. The donor characteristics, cold ischemia times, donor weights, histological evaluations and reasons for transplant non-eligibility are summarized in Supplementary Table S1. The composition of the NMP solution is provided in Supplementary Table S2.

The cold ischemia time was on average 29 h and 20 min, ranging from 16 to 32 h. The weight of the kidneys was on average 368 ± 129 g at the beginning of the perfusion and 377 ± 149 g at the end, with a weight gain of about 9 ± 22.7 g during the perfusion.

During NMP, hemodynamic changes demonstrated a rise in flow and decline in intravascular resistances over time. The mean of flow adjusted to the weight of the kidney was 27.9 ± 9 ml/min/100 g at time 0 and raised to 44 ± 19.9 ml/min/100 g after 2 h. The intravascular resistances decreased from 0.81 ± 0.3 mmHg·min/L at the beginning of the experiment to 0.55 ± 0.13 mmHg·min/L at the end of the procedure. The temperature was stably between 36–37 °C for all the experiments. We observed a great variability in terms of ultrafiltrate production between kidneys. The mean value of ultrafiltration was 0.36 ± 0.59 ml/min in the first 15 min and peaked to 0.66 ± 0.82 ml/min in the following 15 min. It subsequently declined to 0.44 ± 0.46 ml/min until 1 h of perfusion and to 0.28 ± 0.28 ml/min during the last hour (Supplementary Fig. S1).

No significant changes according to Karpinski score were observed at histological examination after NMP (data not shown).

### Protein composition of kidney tissue samples

**Figure 1:**
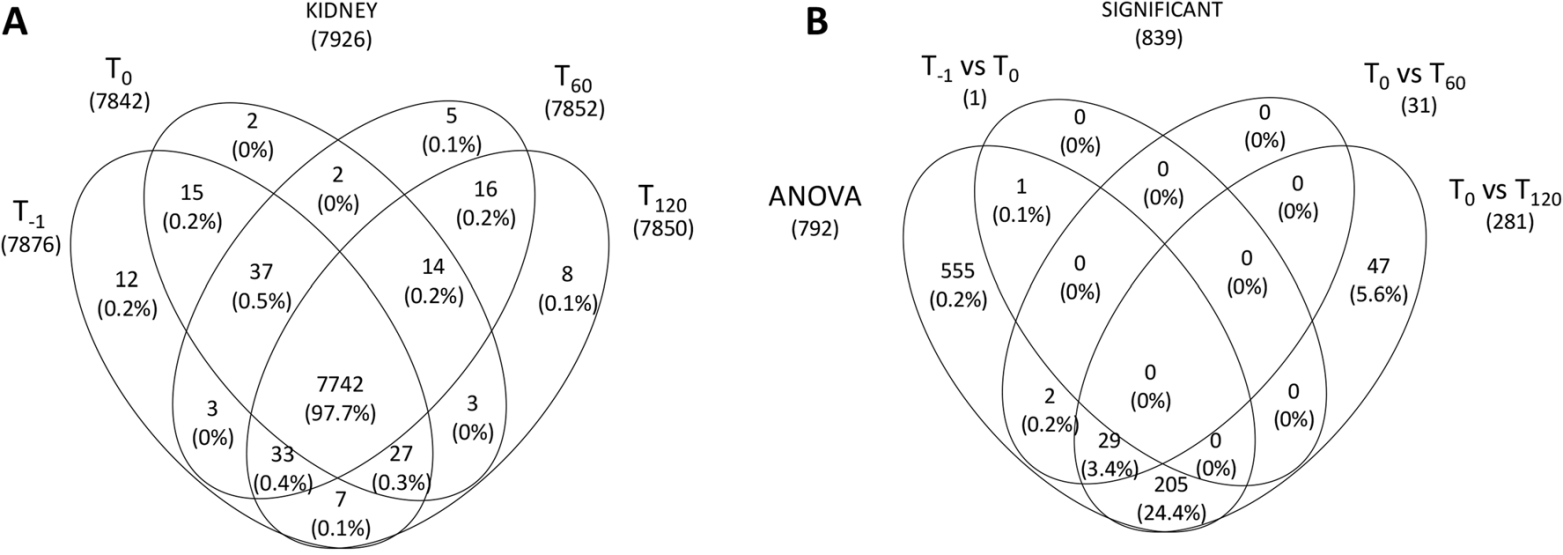
Venn diagram of all proteins identified in kidney tissue samples. **(A)** Venn diagram showing common and exclusive proteins in kidney tissue samples at four time points. The numbers (and percentages) of proteins in the overlapping and non-overlapping areas are indicated. **(B)** Venn diagram showing common and exclusive proteins based on the multivariate and univariate statistical analysis of kidney tissue samples. The numbers (and percentages) of proteins in the overlapping and non-overlapping areas are indicated.

Biopsies of the eight kidneys were analyzed by high-resolution mass spectrometry. This revealed a total of 7926 proteins, 7742 (97.7%) of which were common to all four time points. Notably, 12 (0.2%), 2 (0%), 5 (0.1%) and 8 (0.1%) proteins were exclusively found at time points T-1, T0, T60 and T120, respectively (Fig. 1A).

We applied one-way ANOVA (time series) to identify proteins that changed significantly in abundance during perfusion, and a t-test to identify the proteins that best distinguished between T0 and the other three time points. This revealed 839 significant proteins (according to ANOVA and t-test, and excluding overlapping); 792 differed significantly over time during perfusion by ANOVA. One that differed significantly between T0 and T-1, 31 that differed significantly between T0 and T60, and 281 that differed significantly between T0 and T120 (according to t-test) (Fig. 1B and Supplementary Table S3). The 839 statistically significant proteins included 169 showing a steady increase in abundance during perfusion, 196 showing a steady decrease, and 132 showing no change in abundance.

Volcano plots were used to visualize the univariate statistical analysis (Supplementary Fig. S2).

**Figure 2:**
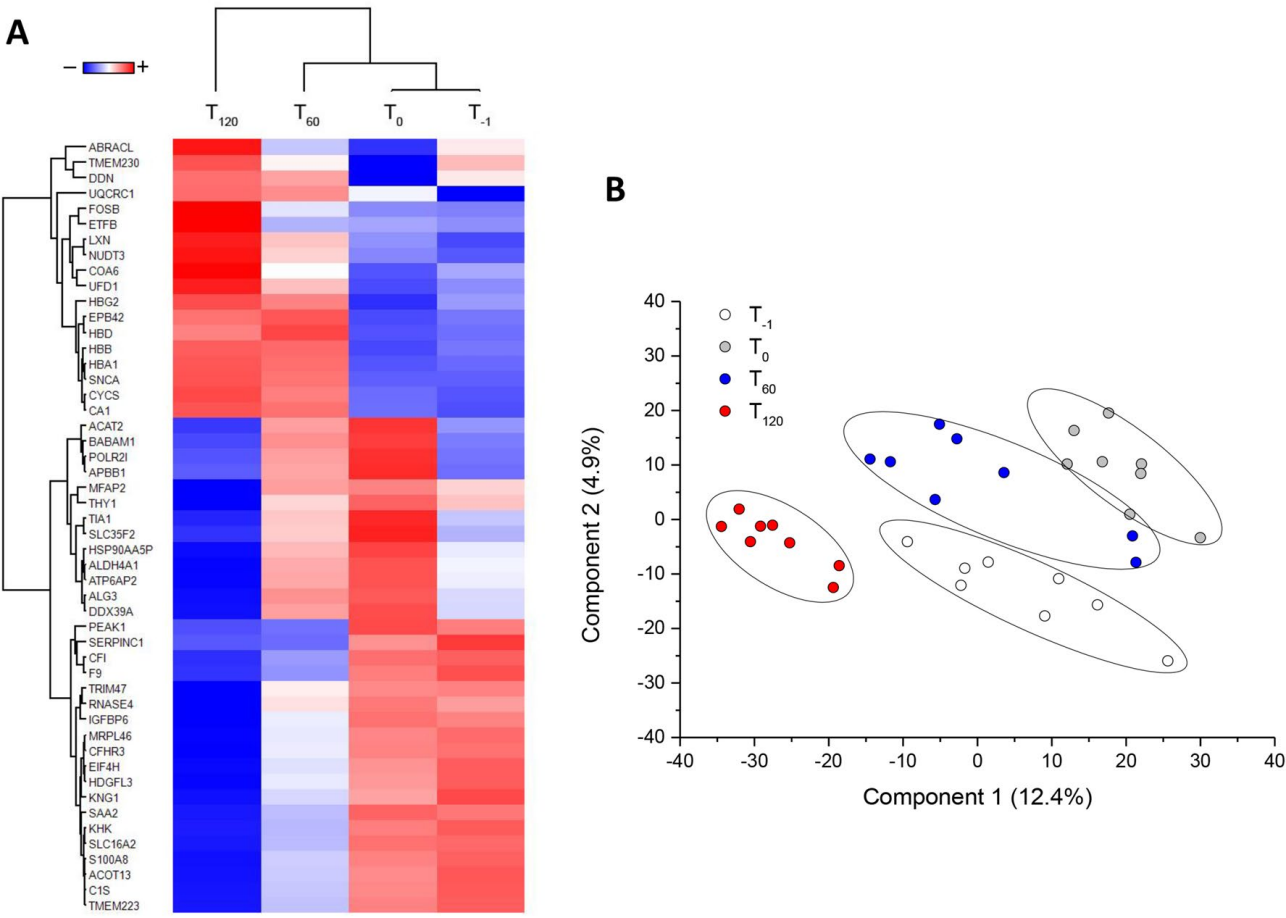
Heat map and partial least squares discriminant analysis of kidney proteins showing the maximum discrimination between the four time-points. **(A)** Heat map of 50 top-ranking proteins identified by statistical analysis. Each row represents a protein and each column corresponds to a time point. Normalized Z-scores of protein abundance are depicted as a pseudocolor scale, with red and blue representing upregulation and downregulation, respectively. The dendrogram shows the outcome of unsupervised hierarchical clustering, placing similar proteome profile values near each other. Visual inspection of the dendrogram and heat map confirms the ability of these proteins to clearly distinguish between the different conditions. **(B)** Two-dimensional scatter plot of PLS-DA representing four time points: T-1 (white circles), T0 (gray circles), T60 (blue circles), and T120 (red circles) based on the combined application of ANOVA, t-test, SVM learning and PLS-DA. Ellipsis indicates 95% confidence interval. Visual inspection of the scatter plot demonstrates the ability of these proteins to clearly distinguish between the different time points.

SVM learning and PLS-DA were used to identify a core panel of ranked proteins that maximized the distinctions between the four time points. Their expression profiles after Z-score normalization were then represented as a heat map (Fig. 2A). Multidimensional scaling indicated the level of discrimination of the selected proteins (Fig. 2B).

GO enrichment analysis revealed that the proteins most strongly upregulated at the end of NMP treatment (T120) were involved in electron transport coupled to ATP synthesis (COA6, UQCRC1, CYCS and ETFB) as well as hydrogen peroxide catabolism and bicarbonate transport (CA1, HBA1, HBB, HBD and HBG2). Some of the proteins significantly downregulated at T120 represented the complement system and coagulation cascades, as well as their regulators (KNG1, F9, C1S, SERPINC1, CFI and CFHR3).

### Protein composition in urine samples

**Figure 3:**
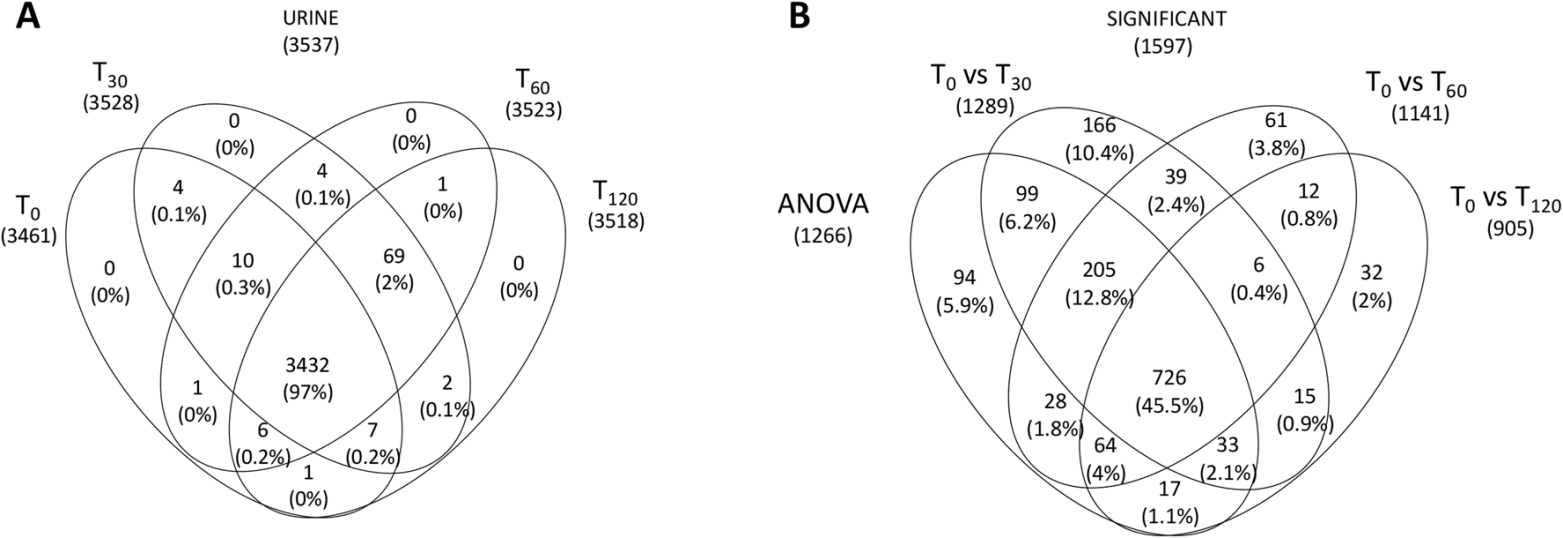
Venn diagram of all proteins identified in urine samples. **(A)** Venn diagram showing common and exclusive proteins in urine samples at four time points. The numbers (and percentages) of proteins in the overlapping and non-overlapping areas are indicated. **(B)** Venn diagram showing common and exclusive proteins based on the multivariate and univariate statistical analysis of urine samples. The numbers (and percentages) of proteins in the overlapping and non-overlapping areas are indicated.

To validate the results from the renal biopsies, we analyzed the urine proteome at four time points. The urine samples of four patients were not included in this analysis because of the large number of erythrocytes. We identified 3537 proteins in total, 3432 (97%) of which were common to all four time points (Fig. 3A). Importantly, 3325 of the urine proteins were also found in the tissue samples.

We applied the one-way ANOVA (time series) to identify the proteins that changed in abundance during perfusion, and a t-test to identify the proteins that best distinguished between T0 and the other three time points. This revealed 1597 significant proteins (according to ANOVA and t-test and excluding overlapping); 1266 differed significantly over time during perfusion (by ANOVA); 1289 that differed significantly between T0 and T30, 1141 that differed significantly between T0 and T60, and 905 that differed significantly between T0 and T120 (Fig. 3B). The 1597 statistically significant proteins included 205 showing a steady increase in abundance during perfusion, nine showing a steady decrease, and 19 showing no change in abundance (Supplementary Table S4). Among the 2323 proteins showing statistically significant differences between time points, 105 (4.5%) were also found in the tissue samples.

**Figure 4:**
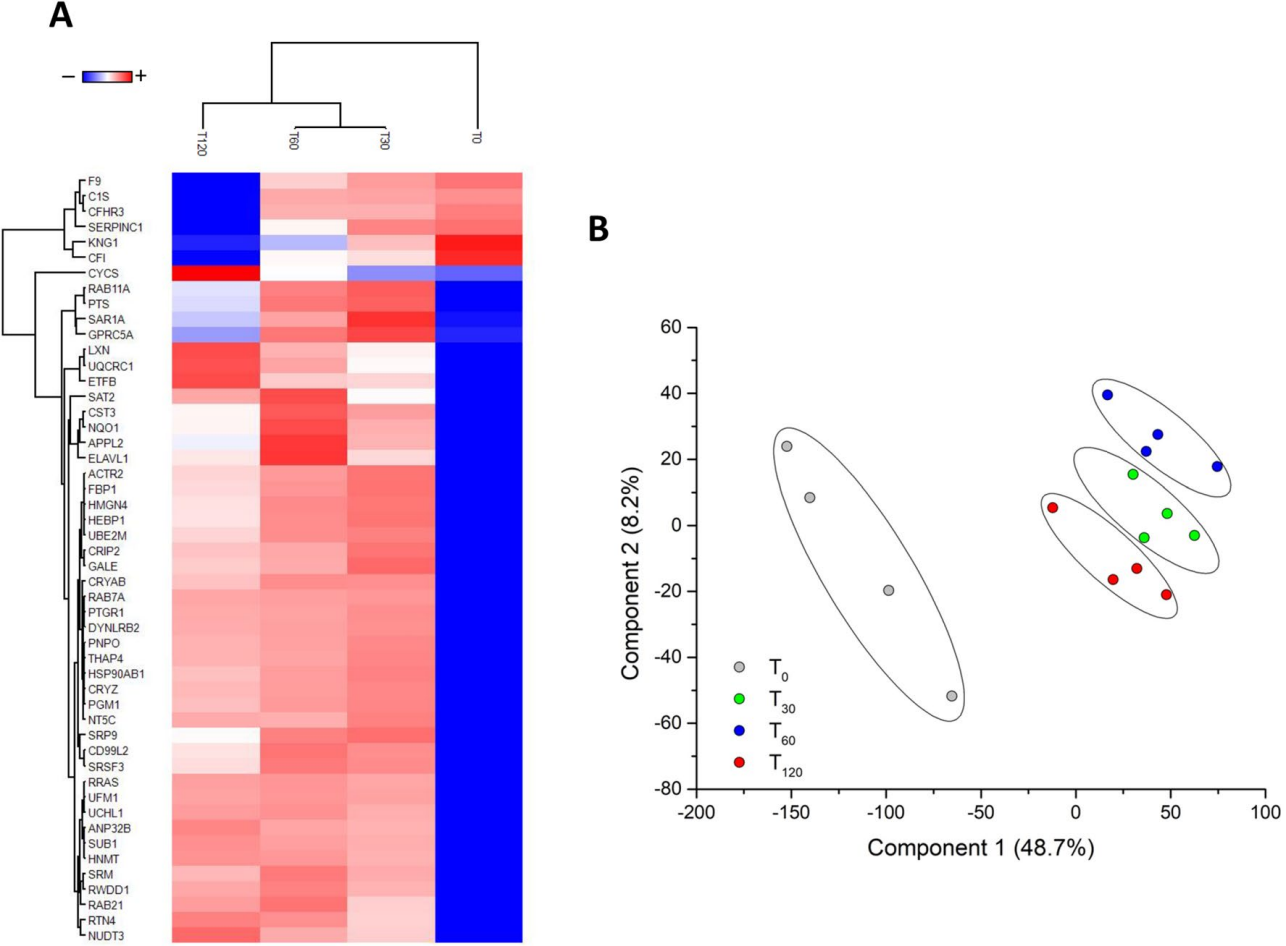
Heat map and partial least squares discriminant analysis of urine proteins showing the maximum discrimination between the four time-points. **(A)** Heat map of 50 top-ranking proteins identified by statistical analysis. Each row represents a protein and each column corresponds to a time point. Normalized Z-scores of protein abundance are depicted as a pseudocolor scale, with red, white and blue representing upregulation, no change and downregulation, respectively. The dendrogram shows the outcome of unsupervised hierarchical clustering, placing similar proteome profile values near each other. Visual inspection of the dendrogram and heat map confirms the ability of these proteins to clearly distinguish among the different conditions. **(B)** Two-dimensional scatter plot of PLS-DA representing four time points: T0 (white circles), T30 (gray circles), T60 (blue circles), and T120 (red circles) based on the combined application of ANOVA, t-test, SVM learning and PLS-DA. Ellipsis indicates 95% confidence interval. Visual inspection of the scatter plot demonstrates the ability of these proteins to clearly distinguish between the different time points.

**Figure 5:**
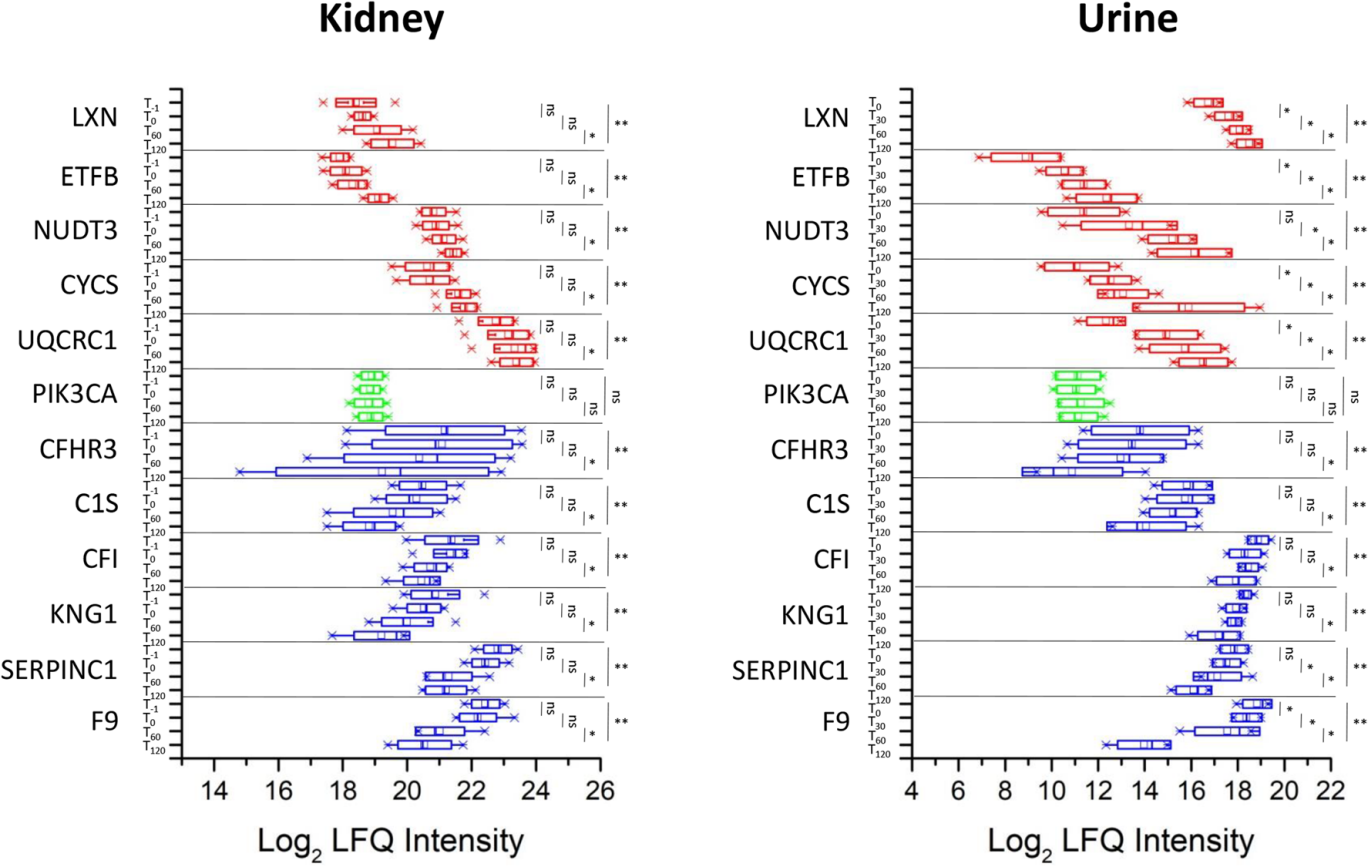
Label-free quantitation intensity box plot of statistically significant proteins shared between the kidney tissue and urine samples. Red, blue and green indicate proteins that increase, decrease or do not change in abundance during normothermic machine perfusion, respectively. * Statistically significant by T-test; ** statistically significant by ANOVA in time series analysis (*p* values are reported in Supplementary Tables S3 and S4).

SVM learning and PLS-DA were used to identify a core panel of ranked proteins that maximized the differences between the four time points. Their expression profiles after Z-score normalization were then represented as a heat map (Fig. 4A). Multidimensional scaling revealed clear discrimination between the cluster representing T0 and the other time points (Fig. 4B). These included five proteins that increased in abundance during perfusion (LXN, ETFB, NUDT3, CYCS and UQCRC1), six that decreased in abundance (CFHR3, C1S, CFI, KNG1, SERPINC1 and F9), and one (PIK3CA) that remained at the same level (Fig. 5). However, Latexin (LXN) resulted the most-upregulated protein at T120 in both urine and tissue samples. The level of expression of each of these 12 top selected proteins (highlighted in grey in the Supplementary Tables S3 and S4) in the kidneys and urine was not significantly correlated to both organ cold ischemia time (CIT) or donors’ serum creatinine levels.

Given that NMP maintains the metabolic activity of kidneys, we looked for proteins involved in fatty acid oxidation and synthesis to discover potential differences across the different time points. Several enzymes were significantly upregulated in urine samples at T120, including alcohol dehydrogenase 1B (ADH1B), 4-trimethylaminobutyraldehyde dehydrogenase (ALDH9A1), α-aminoadipic semialdehyde dehydrogenase (ALDH7A1), long-chain-fatty-acid-CoA ligase 1 (ACSL1), fatty acid synthase (FASN), long-chain-fatty-acid-CoA ligase 5 (ACSL5) (Supplementary Fig. S3). We did not observe the same trend in the kidney samples, but peroxisomal acyl-coenzyme A oxidase 1 (ACOX1) was significantly downregulated following the NMP treatment (Supplementary Fig. S3).

### Gene set enrichment analysis

**Figure 6:**
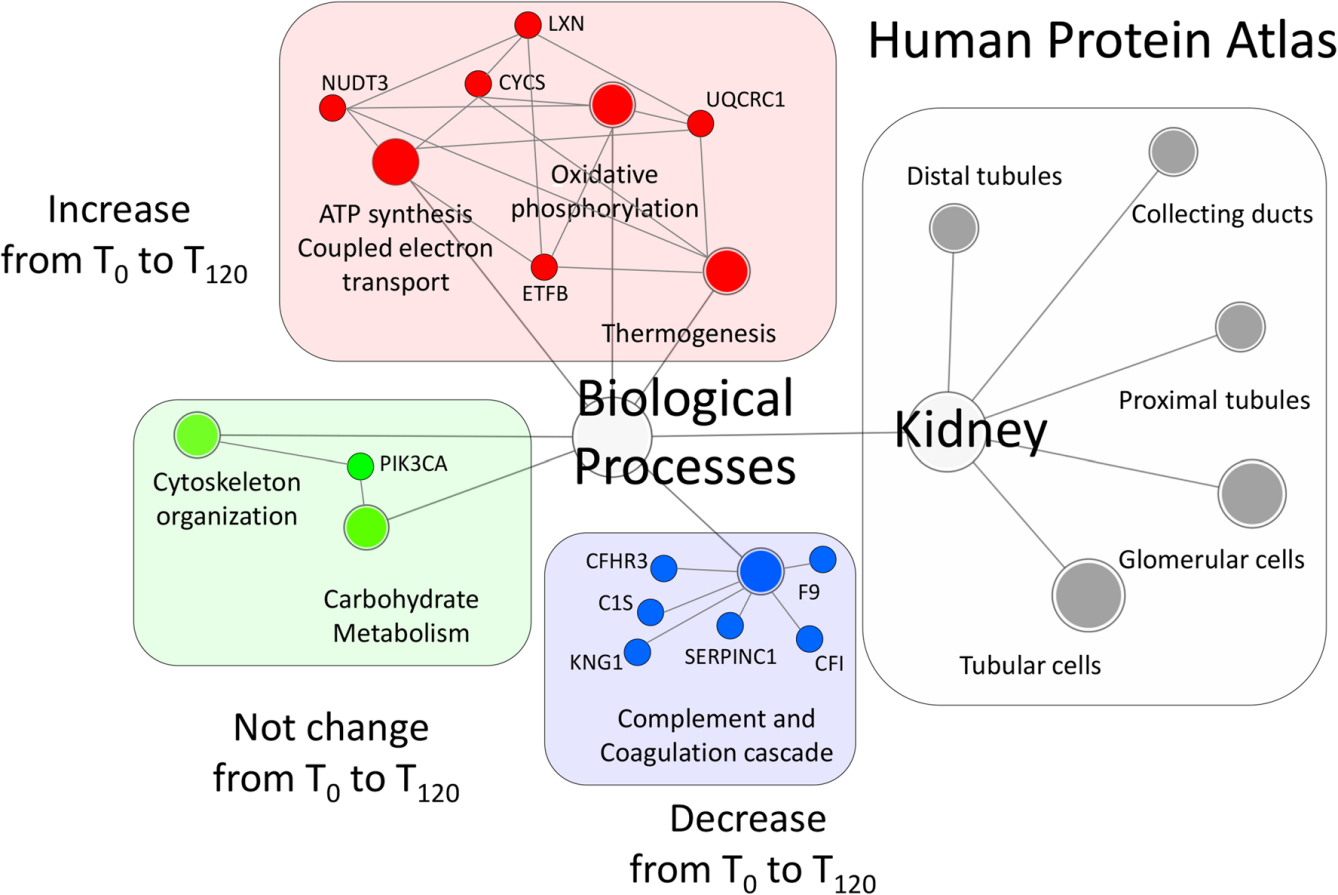
Gene set enrichment analysis network. Nodes and edges represent GO annotation terms and their interactions, respectively. Red, blue and green circles indicate GO annotation terms and proteins that increase, decrease or do not change in abundance during normothermic machine perfusion, respectively.

The diverse expression profiles of kidney proteins during perfusion could indicate functional alterations, so we used GSEA to identify GO annotations enriched among the proteins with statistically significant differences in abundance. In the kidney samples, we identified 119 significantly enriched GO terms, 28 associated with proteins that increased in abundance during perfusion, 15 associated with proteins that decreased in abundance, and seven associated with proteins showing no change (Supplementary Tables S4, S5, S6). The results are summarized in a network diagram (Fig. 6). Among the proteins that increased in abundance during perfusion, GSEA indicated a statistically significant enrichment of GO terms associated with oxidative phosphorylation, thermogenesis, and ATP synthesis coupled to electron transport. Among the proteins that were depleted or unchanged during perfusion, GSEA indicated a statistically significant enrichment of GO terms associated with the complement system, coagulation cascade, cytoskeletal organization and carbohydrate metabolism. Interestingly, using GO annotation terms extracted from the Human Protein Atlas, all statistically significant proteins were enriched for proteins associated with the kidney. The same analysis was applied to the urine samples. GO analysis identified 242 significantly enriched GO terms, 17 of which were associated with proteins that increased in abundance during perfusion, five associated with proteins that decreased in abundance, and three associated with proteins showing no change (Supplementary Tables S7, S8, S9). The 12 proteins that maximize discrimination between the four time points are shown in Fig. 6.

### Validation of mass spectrometry results

**Figure 7:**
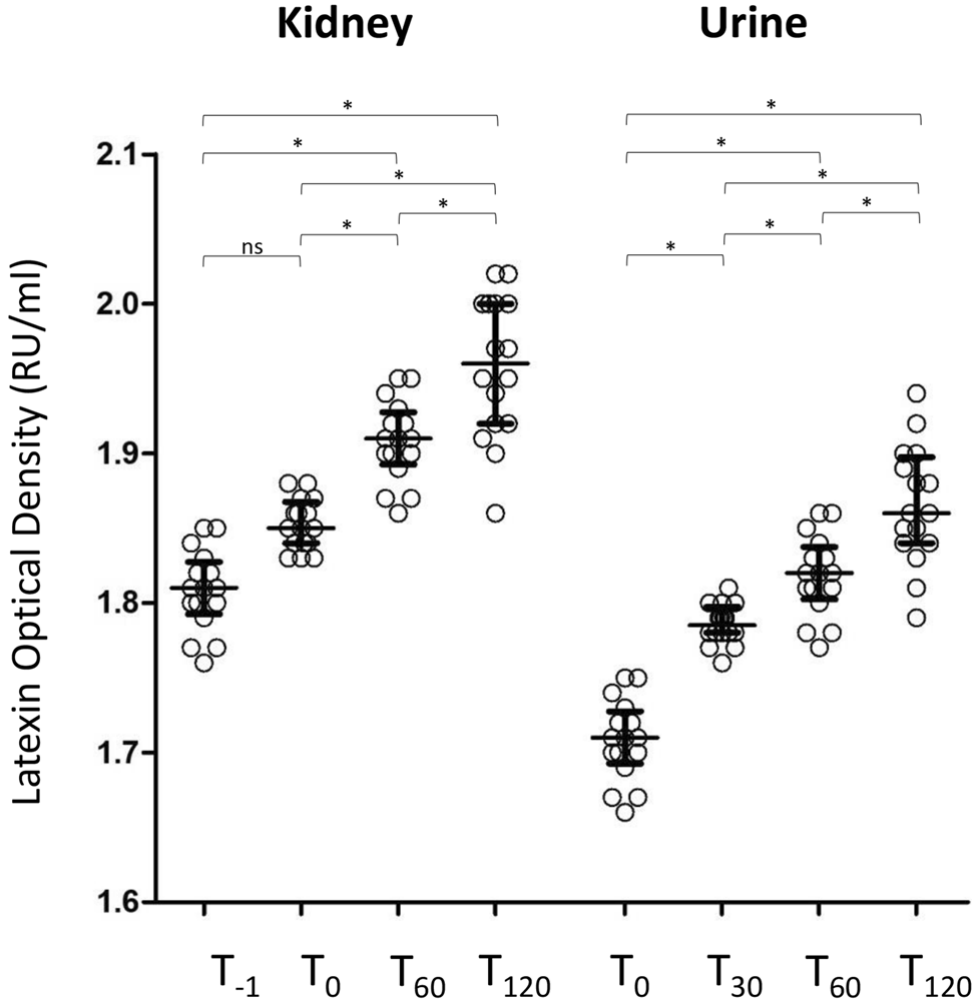
Latexin (LXN) levels in kidney and urine samples. LXN expression levels are measured by direct homemade ELISA in all kidney and urine samples. In both type of samples LXN was significantly increased in a time-dependent manner (p ≤ 0.001). Data are expressed as Relative Unit/ml (RU/ml). n.s.: not significant. *p < 0.0001 by Friedman test with Dunn’s comparison correction.

Then, we used a direct homemade ELISA to measure the protein content of LXN in kidney samples and urine. In both types of samples, LXN expression level was significantly increased (p ≤ 0.0001) in a time-dependent manner (Fig. 7).

## Discussion

Normothermic machine perfusion (NMP) is an alternative to hypothermic preservation that helps to prevent the negative biological effects of I/R injury, particularly in kidneys from ECDs and DCD donors that are more susceptible to delayed graft function and, in some cases, a complete loss of activity. NMP provides oxygen and nutrients while circulating the perfusion solution at a near-physiological temperature, thus restoring and maintaining the metabolic activity of the kidney and replenishing depleted ATP^11–13^. This enhanced preservation method avoids severe morphological and functional alterations that may affect the outcome after transplantation. NMP can also prolong the *ex-situ* preservation time, enabling a more accurate and extended assessment of organ viability before transplantation. However, the biological changes induced by NMP are only partially understood.

To address this knowledge gap, we used an innovative high-resolution mass spectrometry method to reveal changes in the tissue and urinary proteome of marginal kidneys (rejected for transplantation following histological evaluation) during and after NMP treatment for 120 min. Machine learning algorithms (SVM learning and PLS-DA) revealed that a short period of NMP treatment, contrarily to cold static preservation, altered the abundance of many proteins in the kidney tissue and urine samples, with 4.5% of the identified proteins modulated in both types of sample. We found that the proteins upregulated by NMP were primarily involved in oxidative phosphorylation, ATP synthesis coupled to electron transport, and thermogenesis, whereas those downregulated by NMP included key factors and regulators of the complement system and coagulation cascade. The observed effects on oxidative phosphorylation may reflect the re-establishment of oxygen intake and the maintenance of the kidney at a physiological temperature, avoiding the onset of hypoxic-driven acute kidney insults and the development of early chronic organ alterations. Similar results were previously reported following the NMP treatment (with urine recirculation) of discarded human kidneys^14^ and in a porcine auto-transplantation model undergoing normothermic ex vivo kidney perfusion (NEVKP) for 8 h^15^. In these studies, normothermic treatment had a major impact on several biochemical properties and metabolic pathways, including oxidative phosphorylation, fatty acid β-oxidation and the tricarboxylic acid cycle, compared to static cold storage. These responses may facilitate at least a partial recovery from I/R injury by reducing acute morphological and functional alterations and minimizing the early onset of organ fibrosis.

SVM learning and PLS-DA identified five proteins that increased in abundance (LXN, ETFB, NUDT3, CYCS and UQCRC1) and six that decreased in abundance (CFHR3, C1S, CFI, KNG1, SERPINC1, F9) in both urine and kidney tissue after 120 min of NMP. These may be considered key elements of the biological processes induced by NMP and, if validated in further studies, could be developed as biomarkers or therapeutic targets. Some of the upregulated proteins were found to be involved in mitochondrial metabolism and the antioxidant/redox machinery.

Latexin (LXN), also known as tissue or endogenous carboxypeptidase inhibitor (ECI), resulted the most up-regulated protein after 120 min of NMP treatment. Interestingly, this protein is highly expressed in several tissues (including heart, prostate, ovary, kidney, pancreas and colon, brain) where it plays a role in inflammation and cancer (as tumor suppressor)^16–18^. Additionally, it may exert a protective effect on thrombotic renal dysfunction enhancing the fibrinolysis^19^. This effect could mitigate the consequences of the renal I/R which is associated with activation of the coagulation system and accumulation of blood clotting factors in the kidney^20^. Previous studies^21–24^ have shown that renal I/R can cause the accumulation of fibrinogen in the kidney. Fibrinogen is the main protein of the coagulation system and consists of two identical subunits that contain three polypeptide chains: Aα, Bβ, and γ^25^. Besides its important function in coagulation, fibrinogen plays a critical role in inflammation, wound healing, and angiogenesis by interacting with blood cells, endothelial cells, and other cell types after leaking into the extravascular space^26–28^. Although leakage of circulating fibrinogen into areas of acute damage may aggravate injury and trigger inflammation^29,30^ its presence might also be instrumental for normal regeneration^26^. However, although we can suggest a potential positive effect of the up-regulation of Latexin, additional studies are needed to better address this point.

Electron transfer flavoprotein subunit β (ETFB) shuttles electrons between primary flavoprotein dehydrogenases involved in mitochondrial fatty acid/amino acid catabolism and the membrane-bound electron transfer flavoprotein ubiquinone oxidoreductase^31^. Nudix hydrolase 3 (NUDT3) hydrolyzes dinucleotides and inositol pyrophosphates, helping to repair DNA damage and maintain cell survival during oxidative stress^32^. Ubiquinol-cytochrome c reductase core protein 1 (UQCRC1) is a core subunit of mitochondrial respiratory chain complex III, and its upregulation may explain the observed increase in oxidative phosphorylation and ATP production. Cytochrome c (CYCS) is a small protein that plays a key role in the mitochondrial electron transport chain. It associates with the inner membrane of the mitochondrion and accepts electrons from cytochrome b, transferring them to the cytochrome oxidase complex. These results could confirm previous findings that NMP promotes the recovery of mitochondrial function in the kidney and liver, increasing intracellular ATP levels and mitigating the tissue injury that occurs during rewarming^14,33^.

The proteins depleted by NMP treatment included components of the complement system and coagulation cascade, which are involved in the damage to renal tubular cells following I/R injury and in the subsequent inflammatory response^34–36^. NMP was previously shown to partially influence this pathway although with contrasting results^37,38^. Our data suggest that the complement pathway may become hyper-activated (in part compensated by the downregulation of C1s proteases) in response to non-immune insults such as I/R. Complement activation within the injured kidney is also a proximal cause of many downstream inflammatory events in the renal parenchyma that can exacerbate kidney injury^39^. The secreted proteins complement factor H-related protein 3 (CFHR3) and complement factor I (CFI) are essential regulators of the complement cascade.

We found that NMP had a strong impact on coagulation, as demonstrated by the declining levels of kininogen-1 (KNG1), SERPINC1 and F9. KNG is the precursor of the kallikrein-kinin system, which cooperates with coagulation factor XI to form the contact activation system. Kininogen is also the source of the vasoactive nonapeptide bradykinin^40^. SERPINC1 (anti-thrombin III) is an essential plasma protease inhibitor and a member of the serpin superfamily that regulates blood coagulation by inhibiting thrombin and other activated serine proteases in the coagulation system^41^. F9 (vitamin K-dependent coagulation factor IX) is converted to an active form by factor XIa, which excises the activation peptide and thus generates a heavy chain and light chain held together by one or more disulfide bonds. The role of activated factor IX in the coagulation cascade is to activate factor X by interacting with Ca^2+^, membrane phospholipids, and factor VIII^42^.

Metabolic networks related to hypoxia and the complement and coagulation cascades are the major pathways enhanced in organ donors before organ retrieval and the cessation of blood circulation and are therefore the most advantageous targets in the donor to improve long-term allograft survival^43^. The inhibition of complement in brain-dead rats has been shown to improve renal function^44^. The complement activation cascade can be targeted at different levels to attenuate complement-mediated injury by using, for example, soluble complement regulator proteins or antibodies against complement components, their split products, or complement receptor antagonists. Only a few of these agents have been used in patients for indications other than kidney transplantation. Recombinant or plasma-derived C1-esterase inhibitor is currently approved for the routine prophylaxis of hereditary angioedema by blocking the classical and lectin pathways of complement activation, as well as proteases of the fibrinolytic, clotting and kinin pathways^45^.

Our proteomics analysis showed that one group of proteins upregulated by NMP was involved in the acid–base balance, including carbonic anhydrase (CA1), which plays an important role in H^+^ secretion and HCO_3_^−^ reabsorption by kidney tubular cells^46^. This natural preservation of the acid-basic balance, although is in part induced by the sodium bicarbonate in the perfusion solution, may contribute to the preservation of organ function after transplantation^14^. We also found that several enzymes involved in fatty acid oxidation and synthesis were down-regulated in the kidney after 120 min of NMP. These included ACOX1, the first and rate-limiting enzyme of the peroxisomal β-oxidation pathway which uses oxygen as a substrate for fatty acid oxidation and its activity is reduced by both hypoxia and proteolytic degradation during the course of reperfusion^47,48^. It is plausible that the same mechanism may reduce the content of fatty acid oxidation enzymes during short-term NMP following cold ischemia time.

In conclusion, our experiments revealed that NMP for 120 min can induce important metabolic changes that may help to preserve the biochemical functions of suboptimal kidneys. Similar effects on other marginal organs could have a positive impact on their functionality and survival. Our results provide new insights into the molecular responses to NMP and, when validated in larger cohorts of patients, could result in the development of novel biomarkers and therapeutic targets that help clinicians to optimize this treatment and reduce the risk of complications, thus enhancing graft survival.

## Methods

### Source of kidneys and ethical approval

Our study was based on eight kidneys recovered for transplantation but deemed unsuitable according to Nord Italia Transplant program guidelines and a histological evaluation using the Karpinski/Remuzzi score^49^. After transfer to the University-Hospital of Padova, NMP was performed by the clinical staff of the Kidney Transplant Center Unit.

This study was approved by the Ethics Committee of Padova (CESC 4994/AO/21). The study was conducted according to the Helsinki declaration and informed consents were obtained by the family or legal guardian. All experiments were performed in accordance with relevant guidelines and regulations.

### Normothermic machine perfusion

All eight kidneys were perfused using a Kidney Assist device (XVIVO) adapted for NMP. Before cannulating the renal artery and ureter, the kidney was weighed and connected to the machine. Perfusion was started at 37 °C, with a pressure of 50 mmHg, increasing slowly to 70 mmHg in the first 10 min. Before starting NMP, the perfusate was analyzed using an iSTAT blood gas analyzer. Major alterations in electrolytes and the acid–base balance were corrected before connecting the kidney and starting the perfusion, which lasted for 2 h. The ultrafiltrate was reintroduced after collecting a sample and recording the amount as previously described^14^. The kidneys were sent for disposal at the end of the procedure.

### Kidney biopsies and urine samples

We prepared core biopsies of the kidney parenchyma using 18-gauge needles as soon as the organs were deemed unsuitable for transplantation (evaluation time, T-1), and also at the beginning of the experiment (during back table preparation, T0). Further biopsies were taken after 60 min of perfusion (T60) and after 120 min, the end of the treatment (T120). Urine samples were collected at T0 (urine produced in the first 15 min after the beginning of normothermic reperfusion), T30, T60 and T120. They were centrifuged (10,000 × g, 5 min, 4 °C) to remove cells/debris. All samples were stored at − 80 °C until use.

### Preparation of samples for proteomic analysis

Urine samples (100 µl) were denatured, reduced and alkylated in 100 µl iST-LYSE buffer (PreOmics) for 10 min at 95 °C, with a vigorous shaking at 1000 rpm. Renal tissues were washed three times with PBS and lysed with guanidine lysis buffer (6 M guanidine hydrochloride, 10 mM Tris-(2-carboxyethyl) phosphine hydrochloride, 40 mM 2-chloroaceteamide, 100 mM Tris–HCl, pH 8) containing protease and phosphatase inhibitors. The samples were homogenized by applying four cycles of 4 min in a T10 basic ULTRA-TURRAX (IKA). The protein lysates were then heated at 95 °C for 10 min before three cycles of 30 s in a UP200St ultrasonic processor (Hielscher). The protein concentration was estimated using a tryptophan assay^50^.

Urine and renal proteins were isolated using the PAC method^51^ and a Freedom EVO liquid handler (TECAN). Briefly, protein aggregation was induced by adding 70% acetonitrile along with magnetic beads, which were retained when the supernatant was removed. The beads were washed sequentially with acetonitrile, 70% ethanol and isopropanol, then resuspended in 25 mM Tris–HCl (pH 8). The captured proteins were digested overnight at 37 °C with trypsin and LysC at ratios of 1:50 and 1:100 (to the protein content) respectively. The resulting peptides were desalted using Stage-Tips and analyzed by nano-UHPLC-MS/MS using an Ultimate 3000 RSLC coupled to an Orbitrap Fusion Tribrid mass spectrometer with a high-field asymmetric waveform ion mobility spectrometry (FAIMS) Pro Interface (Thermo Fisher Scientific).

### Analysis of renal tissue peptides by nano-UHPLC-MS/MS

Peptides were separated on a 200-cm µPAC C18 column (PharmaFluidics) mounted in a thermostatic column compartment maintained at 50 °C. We applied a gradient of 5–7% buffer B (80% acetonitrile in water, supplemented with 5% DMSO and 0.1% formic acid) at a flow rate of 750 nl/min dropping to 350 nl/min over a period of 12 min. The peptides were then eluted in a non-linear gradient of 7–45% buffer B at a constant flow rate of 350 nl/min for 78 min. The fractions were analyzed in data-independent acquisition (DIA) mode, with Orbitrap detection for MS1 at a resolution of 120,000 within the range 375–1500 m*/z* and an AGC target of 300%. Advanced peak determination was enabled for MS1 measurements. The FAIMS compensation voltage was set to –50 V at standard resolution. Precursors were selected for data-independent fragmentation in 60 windows of 380–980 m*/z* with an overlap of 2 m*/z*. The higher-energy C-trap dissociation (HCD) value was set to 30% and MS2 scans were acquired at a resolution of 15,000 with a maximum injection time (max. IT) of 22 ms and an AGC target of 1000%.

Raw data were processed using FragPipe v17.1 with MS Fragger v3.4^52^ in the DIA_SpecLib_Quant workflow^53^ to build spectral libraries, and DIA-NN v1.8 for DIA quantification. All default settings were applied except the RT Lowes fraction, which was modified to 0.01 for spectral library generation. We screened the UniProt human protein sequence database (release UP000005640_9606 July 2021) and a database of common contaminant proteins.

### Analysis of urine peptides by nano-UHPLC-MS/MS

Peptides were separated as above, but using a 1–7% initial gradient of buffer B. The fractions were analyzed in data-dependent acquisition (DDA) mode with the same resolution and *m/z* range as stated above for the analysis of renal tissue proteins. The top 10 precursors were selected for MS2 analysis. MS/MS spectra were acquired in the linear ion trap (rapid scan mode) after HCD with a collision energy of 30% and a custom AGC target. We applied quadrupole isolation with a 1.6 m*/z* window, dynamic exclusion set to 50 s, and a max. IT of 35 ms.

Raw data were processed using MaxQuant v2.0.3.0^54^. A false discovery rate of 0.01 was used for the identification of proteins, peptides and peptide-spectrum matches. A minimum of seven amino acids was required for peptide identification. The Andromeda engine in MaxQuant was used to search MS/MS spectra against the UniProt human database (release UP000005640_9606 July 2021). N-terminal acetylation, methionine oxidation and asparagine/glutamine deamidation were selected as variable modifications, and carbamidomethyl cysteine was selected as a fixed modification. Quantification intensities were calculated using the default fast MaxLFQ algorithm with the activated option “match between runs.” Raw data processed by MaxQuant were further processed using IceR^55^ with default settings, to reduce the number of missing values.

### Direct ELISA

To confirm the results obtained by LC/MS–MS for LXN expression in kidney and urine samples, homemade direct ELISA was performed. Specificity of the antibody was validated by western blot (Supplementary Fig. S4). Briefly, 5 mg of solubilized human kidney samples and urine at each time point were added to the 96-well Nunc MaxiSorp plate (ThermoFisher Scientific, Waltham, MA, USA) and incubated overnight at 4 °C. After one wash with PBS, the plate was saturated over night at 4 °C with 3% w/v BSA in PBS and subsequently incubated overnight with policlonal anti-human LXN (1:1000) in 3% w/v BSA in PBS-T (PBS with 0.05% v/v Tween-20). After three washes with PBS-T, plate was incubated with secondary antibody HRP conjugated for 2 h at room temperature and the reaction was developed and stopped respectively with TMB substrate and 1 M of sulfuric acid solutions. The absorbance was read at 450 nm in an iMark microplate reader (Bio-Rad, Hercules, CA, USA).

### Statistical analysis

All statistical tests were performed using Origin Lab V9 and the latest version of the software package R available at the time of the experiments. After normalization and missing value imputation with a normal distribution, MS data were analyzed by unsupervised hierarchical clustering using multidimensional scaling (MDS) with k-means and Spearman’s correlation to identify outliers and the dissimilarity between samples. Proteins differing in abundance over the four time points were assessed by one-way analysis of variance (ANOVA) for time series, whereas a t-test was used to identify proteins differing in abundance between T0 and the other three time points. Finally, we used machine learning to identify proteins that maximize discrimination between the four time points, specifically non-linear support vector machine (SVM) learning, and partial least squares discriminant analysis (PLS-DA). For the t-test, differences in protein abundance between two conditions were considered significant with a power of 80% and an adjusted p-value ≤ 0.05 after correction for multiple interactions (Benjamini-Hochberg) and a fold change ≥ 2. Volcano plots were used to quickly visualize statistical differences and the cutoff lines were established using the function y = c/(x–x_0_). The Bland–Altman (MA) plot was used to identify proteins whose abundance did not change during perfusion and the cutoff value for each protein was established using the function x ≤ mean ± √δ^2^/n. In SVM learning, fourfold cross-validation was applied to estimate the prediction and classification accuracy. The whole matrix was randomly divided into two parts, one for learning (65%) and the other to verify the accuracy of the prediction (35%). SVM and PLS-DA generate rank and VIP (variable importance in projection) scores, respectively, to establish a priority list of proteins that distinguish the four time points.

The g:Profiler website (https://biit.cs.ut.ee/gprofiler)^56^ was used to build a functional protein network based on Gene Ontology (GO) annotations and for gene set enrichment analysis (GSEA) using Fisher’s exact test. Significant proteins were uploaded as a gene list with the official gene names as identifiers and *Homo sapiens* as the organism. GSEA results were uploaded and visualized with Cytoscape.

To assess the difference in the levels of LXN between each time point of kidney and urine samples in the direct homemade ELISA, the Friedman test for paired samples with Dunn’s multiple comparison test correction was used. Results were reported as medians and the interquartile range (IQr). Two-sided p values ≤ 0.05 was considered statistically significant.

## Supplementary Information

Supplementary Information 1.

Supplementary Information 2.

Supplementary Information 3.

Supplementary Information 4.

Supplementary Information 5.

Supplementary Information 6.

Supplementary Information 7.

Supplementary Information 8.

Supplementary Information 9.

Supplementary Information 10.

Supplementary Information 11.

Supplementary Information 12.

Supplementary Information 13.

Supplementary Information 14.

## Data Availability

The MS data have been deposited to the ProteomeXchange Consortium (http://www.proteomexchange.org/) via the PRIDE partner repository (dataset identifier PXD036432).

## References

[1] Vanholder R, Domínguez-Gil B, Busic M, Cortez-Pinto H, Craig JC, Jager KJ, Mahillo B, Stel VS, Valentin MO, Zoccali C, Oniscu GC (2021). Organ donation and transplantation: A multi-stakeholder call to action. Nat. Rev. Nephrol..

[2] Aubert O, Kamar N, Vernerey D, Viglietti D, Martinez F, Duong-Van-Huyen JP, Eladari D, Empana JP, Rabant M, Verine J, Rostaing L, Congy N, Guilbeau-Frugier C, Mourad G, Garrigue V, Morelon E, Giral M, Kessler M, Ladrière M, Delahousse M, Glotz D, Legendre C, Jouven X, Lefaucheur C, Loupy A (2015). Long term outcomes of transplantation using kidneys from expanded criteria donors: Prospective, population based cohort study. BMJ.

[3] Summers DM, Watson CJ, Pettigrew GJ, Johnson RJ, Collett D, Neuberger JM, Bradley JA (2015). Kidney donation after circulatory death (DCD): State of the art. Kidney Int..

[4] Fernández AR, Sánchez-Tarjuelo R, Cravedi P, Ochando J, López-Hoyos M (2020). Review: Ischemia reperfusion injury-a translational perspective in organ transplantation. Int. J. Mol. Sci..

[5] Salvadori M, Rosso G, Bertoni E (2015). Update on ischemia-reperfusion injury in kidney transplantation: Pathogenesis and treatment. World J. Transplant..

[6] Jochmans I, O'Callaghan JM, Pirenne J, Ploeg RJ (2015). Hypothermic machine perfusion of kidneys retrieved from standard and high-risk donors. Transpl. Int..

[7] Hansson J, Mjörnstedt L, Lindnér P (2018). The risk of graft loss 5 years after kidney transplantation is increased if cold ischemia time exceeds 14 h. Clin. Transplant..

[8] Elliott TR, Nicholson ML, Hosgood SA (2021). Normothermic kidney perfusion: An overview of protocols and strategies. Am. J. Transplant..

[9] Weissenbacher A, Huang H, Surik T, Lo Faro ML, Ploeg RJ, Coussios CC, Friend PJ, Kessler BM (2021). Urine recirculation prolongs normothermic kidney perfusion via more optimal metabolic homeostasis-a proteomics study. Am. J. Transplant..

[10] Woud WW, Arykbaeva AS, Alwayn IPJ, Baan CC, Minnee RC, Hoogduijn MJ, Boer K (2022). Extracellular vesicles released during normothermic machine perfusion are associated with human donor kidney characteristics. Transplantation.

[11] Hosgood SA, van Heurn E, Nicholson ML (2015). Normothermic machine perfusion of the kidney: Better conditioning and repair?. Transpl. Int..

[12] Jochmans I, Akhtar MZ, Nasralla D, Kocabayoglu P, Boffa C, Kaisar M, Brat A, O'Callaghan J, Pengel LH, Knight S, Ploeg RJ (2016). Past, present, and future of dynamic kidney and liver preservation and resuscitation. Am. J. Transplant..

[13] De Beule J, Jochmans I (2020). Kidney perfusion as an organ quality assessment tool-are we counting our chickens before they have hatched?. J. Clin. Med..

[14] Weissenbacher A, Lo Faro L, Boubriak O, Soares MF, Roberts IS, Hunter JP, Voyce D, Mikov N, Cook A, Ploeg RJ, Coussios CC, Friend PJ (2019). Twenty-four-hour normothermic perfusion of discarded human kidneys with urine recirculation. Am. J. Transplant..

[15] McEvoy CM, Clotet-Freixas S, Tokar T, Pastrello C, Reid S, Batruch I, RaoPeters AAE, Kaths JM, Urbanellis P, Farkona S, Van JAD, Urquhart BL, John R, Jurisica I, Robinson LA, Selzner M, Konvalinka A (2021). Normothermic ex-vivo kidney perfusion in a porcine auto-transplantation model preserves the expression of key mitochondrial proteins: An unbiased proteomics analysis. Mol. Cell Proteomics..

[16] Liu Q, Yu L, Gao J, Fu Q, Zhang J, Zhang P, Chen J, Zhao S (2000). Cloning, tissue expression pattern and genomic organization of Latexin, a human homologue of rat carboxypeptidase A inhibitor. Mol. Biol. Rep..

[17] Burkard TR, Planyavsky M, Kaupe I, Breitwieser FP, Bürckstümmer T, Bennett KL, Superti-Furga G, Colinge J (2011). Initial characterization of the human central proteome. BMC Syst. Biol..

[18] Ni QF, Tian Y, Kong LL, Lu YT, Ding WZ, Kong LB (2014). Latexin exhibits tumor suppressor potential in hepatocellular carcinoma. Oncol. Rep..

[19] Muto Y, Suzuki K, Iida H, Ishii H (2007). EF6265, a novel plasma carboxypeptidase B inhibitor, protects against renal dysfunction in rat thrombotic glomerulonephritis through enhancing fibrinolysis. Nephron Exp. Nephrol..

[20] Sörensen-Zender I, Rong S, Susnik N, Lange J, Gueler F, Degen JL, Melk A, Haller H, Schmitt R (2013). Role of fibrinogen in acute ischemic kidney injury. Am. J. Physiol. Renal. Physiol..

[21] Ajay AK, Saikumar J, Bijol V, Vaidya VS (2012). Heterozygosity for fibrinogen results in efficient resolution of kidney ischemia reperfusion injury. PLoS ONE.

[22] Eneström S, Druid H, Rammer L (1988). Fibrin deposition in the kidney in post-ischaemic renal damage. Br. J. Exp. Pathol..

[23] Frank RD, Schabbauer G, Holscher T, Sato Y, Tencati M, Pawlinski R, Mackman N (2005). The synthetic pentasaccharide fondaparinux reduces coagulation, inflammation and neutrophil accumulation in kidney ischemia-reperfusion injury. J. Thromb. Haemost..

[24] Sevastos J, Kennedy SE, Davis DR, Sam M, Peake PW, Charlesworth JA, Mackman N, Erlich JH (2007). Tissue factor deficiency and PAR-1 deficiency are protective against renal ischemia reperfusion injury. Blood.

[25] Herrick S, Blanc-Brude O, Gray A, Laurent G (1999). Fibrinogen. Int. J. Biochem. Cell Biol..

[26] Drew AF, Liu H, Davidson JM, Daugherty CC, Degen JL (2001). Wound-healing defects in mice lacking fibrinogen. Blood.

[27] Gardiner EE, D'Souza SE (1997). A mitogenic action for fibrinogen mediated through intercellular adhesion molecule-1. J. Biol. Chem..

[28] Laurens N, Koolwijk P, de Maat MP (2006). Fibrin structure and wound healing. J. Thromb. Haemost..

[29] Flick MJ, LaJeunesse CM, Talmage KE, Witte DP, Palumbo JS, Pinkerton MD, Thornton S, Degen JL (2007). Fibrin(OGEN) exacerbates inflammatory joint disease through a mechanism linked to the integrin alphaMbeta2 binding motif. J. Clin. Invest..

[30] Schachtrup C, Lu P, Jones LL, Lee JK, Lu J, Sachs BD, Zheng B, Akassoglou K (2007). Fibrinogen inhibits neurite outgrowth via beta 3 integrin-mediated phosphorylation of the EGF receptor. Proc. Natl. Acad. Sci. U.S.A..

[31] Henriques BJ, Katrine Jentoft Olsen R, Gomes CM, Bross P (2021). Electron transfer Flavoprotein and its role in mitochondrial energy metabolism in health and disease. Gene.

[32] Samper-Martín B, Sarrias A, Lázaro B, Pérez-Montero M, Rodríguez-Rodríguez R, Ribeiro MPC, Bañón A, Wolfgeher D, Jessen HJ, Alsina B, Clotet J, Kron SJ, Saiardi A, Jiménez J, Bru S (2021). Polyphosphate degradation by Nudt3-Zn^2+^ mediates oxidative stress response. Cell Rep..

[33] Parente A, Osei-Bordom DC, Ronca V, Perera MTPR, Mirza D (2020). Organ restoration with normothermic machine perfusion and immune reaction. Front. Immunol..

[34] Danobeitia JS, Djamali A, Fernandez LA (2014). The role of complement in the pathogenesis of renal ischemia-reperfusion injury and fibrosis. Fibrogenesis Tissue Repair..

[35] Peng Q, Li K, Smyth LA, Xing G, Wang N, Meader L, Lu B, Sacks SH, Zhou W (2012). C3a and C5a promote renal ischemia-reperfusion injury. J. Am. Soc. Nephrol..

[36] de Vries B, Köhl J, Leclercq WK, Wolfs TG, van Bijnen AA, Heeringa P, Buurman WA (2003). Complement factor C5a mediates renal ischemia-reperfusion injury independent from neutrophils. J. Immunol..

[37] Hameed AM, Lu DB, Patrick E, Xu B, Hu M, Chew YV, Keung K, P’ng CH, Gaspi R, Zhang C, Robertson P, Alexander S, Thomas G, Laurence J, De Roo R, Wong G, Miraziz R, O'Grady G, Yuen L, Hawthorne WJ, Rogers NM, Pleass HC (2019). Brief normothermic machine perfusion rejuvenates discarded human kidneys. Transpl. Direct..

[38] Jager NM, Venema LH, Arykbaeva AS, Meter-Arkema AH, Ottens PJ, van Kooten C, Mollnes TE, Alwayn IPJ, Leuvenink HGD, Pischke SE, PROPER study consortium (2022). Complement is activated during normothermic machine perfusion of porcine and human discarded kidneys. Front. Immunol..

[39] McCullough JW, Renner B, Thurman JM (2013). The role of the complement system in acute kidney injury. Semin Nephrol..

[40] Ponczek MB (2021). High molecular weight Kininogen: A review of the structural literature. Int. J. Mol. Sci..

[41] Li W, Johnson DJ, Adams TE, Pozzi N, De Filippis V, Huntington JA (2010). Thrombin inhibition by serpins disrupts exosite II. J. Biol. Chem..

[42] Osterud B, Rapaport SI (1977). Activation of factor IX by the reaction product of tissue factor and factor VII: Additional pathway for initiating blood coagulation. Proc. Natl. Acad. Sci. U.S.A..

[43] Damman J, Bloks VW, Daha MR, van der Most PJ, Sanjabi B, van der Vlies P, Snieder H, Ploeg RJ, Krikke C, Leuvenink HG, Seelen MA (2015). Hypoxia and complement-and-coagulation pathways in the deceased organ donor as the major target for intervention to improve renal allograft outcome. Transplantation.

[44] Damman J, Hoeger S, Boneschansker L, Theruvath A, Waldherr R, Leuvenink HG, Ploeg RJ, Yard BA, Seelen MA (2011). Targeting complement activation in brain-dead donors improves renal function after transplantation. Transpl. Immunol..

[45] Davis AE, Mejia P, Lu F (2008). Biological activities of C1 inhibitor. Mol. Immunol..

[46] Koeppen BM (2009). The kidney and acid-base regulation. Adv. Physiol. Educ..

[47] Simon N, Hertig A (2015). Alteration of fatty acid oxidation in tubular epithelial cells: From acute kidney injury to renal fibrogenesis. Front. Med. (Lausanne)..

[48] Gulati S, Ainol L, Orak J, Singh AK, Singh I (1993). Alterations of peroxisomal function in ischemia-reperfusion injury of rat kidney. Biochim. Biophys. Acta..

[49] Remuzzi G, Cravedi P, Perna A, Dimitrov BD, Turturro M, Locatelli G, Rigotti P, Baldan N, Beatini M, Valente U, Scalamogna M, Ruggenenti P, Dual Kidney Transplant Group (2006). Long-term outcome of renal transplantation from older donors. N. Engl. J. Med..

[50] Wiśniewski JR, Gaugaz FZ (2015). Fast and sensitive total protein and Peptide assays for proteomic analysis. Anal Chem..

[51] Batth TS, Tollenaere MX, Rüther P, Gonzalez-Franquesa A, Prabhakar BS, Bekker-Jensen S, Deshmukh AS, Olsen JV (2019). Protein Aggregation capture on microparticles enables multipurpose proteomics sample preparation. Mol. Cell Proteomics..

[52] Kong AT, Leprevost FV, Avtonomov DM, Mellacheruvu D, Nesvizhskii AI (2017). MSFragger: ultrafast and comprehensive peptide identification in mass spectrometry-based proteomics. Nat. Methods..

[53] Brunner AD, Thielert M, Vasilopoulou C, Ammar C, Coscia F, Mund A, Hoerning OB, Bache N, Apalategui A, Lubeck M, Richter S, Fischer DS, Raether O, Park MA, Meier F, Theis FJ, Mann M (2022). Ultra-high sensitivity mass spectrometry quantifies single-cell proteome changes upon perturbation. Mol. Syst. Biol..

[54] Cox J, Mann M (2008). MaxQuant enables high peptide identification rates, individualized p.p.b.-range mass accuracies and proteome-wide protein quantification. Nat. Biotechnol..

[55] Kalxdorf M, Müller T, Stegle O, Krijgsveld J (2021). IceR improves proteome coverage and data completeness in global and single-cell proteomics. Nat. Commun..

[56] Raudvere U, Kolberg L, Kuzmin I, Arak T, Adler P, Peterson H, Vilo J (2019). g:Profiler: A web server for functional enrichment analysis and conversions of gene lists (2019 update). Nucleic Acids Res..

