# Supplementary material for: Proteomics reveals specific biological changes induced by the normothermic machine perfusion of donor kidneys with a significant up-regulation of Latexin
Source: Sci Rep. 2023 Apr 11;13:5920. doi: 10.1038/s41598-023-33194-z (PMC10090051; doi:10.1038/s41598-023-33194-z)
Supplement: Supplementary file 1 — Supplementary Information 1. [file 41598_2023_33194_MOESM1_ESM.docx]

**Figure S1. Perfusion and functional parameters following normothermic machine perfusion. (A)** Flow, **(B)** intra-renal resistance and **(C)** ultrafiltrate production during normothermic machine perfusion. The gray dotted lines represent the single experiment, while the bold black line traces the mean value.
