# Supplementary material for: Proteomics reveals specific biological changes induced by the normothermic machine perfusion of donor kidneys with a significant up-regulation of Latexin
Source: Sci Rep. 2023 Apr 11;13:5920. doi: 10.1038/s41598-023-33194-z (PMC10090051; doi:10.1038/s41598-023-33194-z)
Supplement: Supplementary file 2 — Supplementary Information 2. [file 41598_2023_33194_MOESM2_ESM.docx]

**Figure S2.** **Volcano plot of univariate statistical analysis of proteomics in kidney samples.** The plots are based on the fold change (log_2_) and the P‑value (–log_10_) of all proteins identified in all samples. Volcano plot for **(A)** T-1 vs T0; **(B)** T0 vs T60; **(C)** T0 vs 120. Black circles represent unchanged proteins, red and blue represent the upregulated and downregulated proteins, respectively.
