# Supplementary material for: Proteomics reveals specific biological changes induced by the normothermic machine perfusion of donor kidneys with a significant up-regulation of Latexin
Source: Sci Rep. 2023 Apr 11;13:5920. doi: 10.1038/s41598-023-33194-z (PMC10090051; doi:10.1038/s41598-023-33194-z)
Supplement: Supplementary file 3 — Supplementary Information 3. [file 41598_2023_33194_MOESM3_ESM.docx]

**Figure S3. Beta oxidation and fatty acid synthesis pathway analysis.** Log2 fold change of Label-Free quantitation intensity between time T0 and T120 of beta-oxidation and fatty acid synthesis enzymes identified in the urine and kidney samples. Data were reported as mean (gray square) and standard deviation.
