# Supplementary material for: Proteomics reveals specific biological changes induced by the normothermic machine perfusion of donor kidneys with a significant up-regulation of Latexin
Source: Sci Rep. 2023 Apr 11;13:5920. doi: 10.1038/s41598-023-33194-z (PMC10090051; doi:10.1038/s41598-023-33194-z)
Supplement: Supplementary file 4 — Supplementary Information 4. [file 41598_2023_33194_MOESM4_ESM.docx]

**Western Blot for Latexin**

To evaluate the specificity of anti-human Latexin (LXN) 5 mg of solubilized human kidney samples and urine pooled for each time point for all 8 enrolled organs were separated by sodium dodecyl sulfate polyacrylamide gel electrophoresis (SDS-PAGE) and then transferred to a nitrocellulose membrane. The full-length membrane was blocked, rinsed, labeled, and detected with policlonal anti-human LXN diluted in 3% w/v bovine serum albumin (BSA) in PBS containing 0.05% v/v Tween-20 (PBS-T). After rinsing in PBS-T, the membrane was incubated with HRP-conjugated secondary antibodies (diluted 1:10,000 in 1% w/v BSA in PBS-T). The chemiluminescence signal was acquired and quantified using ChemiDoc and Quantity One software (Bio-Rad, Hercules, CA, USA).

**Figure S4. Western blot for Latexin (LXN).** Representative western blot analysis to validate that the primary antibody is specific for its target and can be used for homemade ELISA in kidney samples and urine. Western blot shows for each polled time point sample a unique band at the molecular weight of LXN that increased in a time-dependent manner.
