# Supplementary material for: Proteomics reveals specific biological changes induced by the normothermic machine perfusion of donor kidneys with a significant up-regulation of Latexin
Source: Sci Rep. 2023 Apr 11;13:5920. doi: 10.1038/s41598-023-33194-z (PMC10090051; doi:10.1038/s41598-023-33194-z)
Supplement: Supplementary file 5 — Supplementary Information 5. [file 41598_2023_33194_MOESM5_ESM.docx]

**Table S1.** **Characteristics of the kidneys undergoing proteomics after normothermic machine perfusion**

| **Donor Age (years)** | **Donor Gender** | **Donor weight (Kg)** | **Cause of donor brain death** | **Serum Creatinine (mg/dL)** | **Biopsy Karpinski Score** | **CIT (h)** | **Reason for Discard** |
| --- | --- | --- | --- | --- | --- | --- | --- |
| 68 | F | 60 | Intracerebral hemorrhage | 1.22 | 7 | 26.8 | Biopsy Findings |
| 77 | M | 85 | Intracerebral hemorrhage | 1.05 | 6 | 28.4 | Biopsy Findings |
| 77 | M | 85 | Intracerebral hemorrhage | 1.05 | 8 | 29.5 | Biopsy Findings |
| 60 | M | 115 | Head trauma | 1.48 | 7 | 28.7 | Biopsy Findings |
| 58 | M | 98 | Intracerebral hemorrhage | 1.23 | 6 | 32.0 | Biopsy Findings and prolonged CIT |
| 72 | F | 68 | Intracerebral hemorrhage | 0.56 | 6 | 32.3 | Biopsy Findings and prolonged CIT |
| 82 | M | 85 | Head trauma | 0.99 | 6 | 32.3 | Biopsy Findings and prolunged CIT |
| 85 | M | 85 | Intracerebral hemorrhage | 0.79 | 6 | 16.7 | Biopsy Findings |
