# Supplementary material for: Proteomics reveals specific biological changes induced by the normothermic machine perfusion of donor kidneys with a significant up-regulation of Latexin
Source: Sci Rep. 2023 Apr 11;13:5920. doi: 10.1038/s41598-023-33194-z (PMC10090051; doi:10.1038/s41598-023-33194-z)
Supplement: Supplementary file 6 — Supplementary Information 6. [file 41598_2023_33194_MOESM6_ESM.docx]

**Table S2.** **Composition of the perfusion solution**

| **Constituents** | **Volume** |
| --- | --- |
| RBCs | 250-350 mL |
| Ringer’s solution | 450 mL |
| Mannitol 15% | 10 mL |
| Glucose 5% | 7 mL |
| Heparin 1000 U/mL | 4 mL |
| Cefazoline 1 g | 10 mL |
| Humalog | 5 IU |
| Dexamethasone 4 mg | 2 mL |
| Cernevit multivitamins | 5 mL |
| Verapamil 5 mg | 5 mL (2 mL bolus at the beginning of perfusion and afterwards 1 mL every 30 minutes) |
| Sodium Bicarbonate 8.4% | 7 mL |
| Calcium gluconate | 5 mL |
